# Modulation of Endotoxicity of *Shigella* Generalized Modules for Membrane Antigens (GMMA) by Genetic Lipid A Modifications: RELATIVE ACTIVATION OF TLR4 AND TLR2 PATHWAYS IN DIFFERENT MUTANTS*

**DOI:** 10.1074/jbc.M114.566570

**Published:** 2014-07-14

**Authors:** Omar Rossi, Isabella Pesce, Carlo Giannelli, Susanna Aprea, Mariaelena Caboni, Francesco Citiulo, Sara Valentini, Ilaria Ferlenghi, Calman Alexander MacLennan, Ugo D'Oro, Allan Saul, Christiane Gerke

**Affiliations:** From the ‡ Novartis Vaccines Institute for Global Health and; § Novartis Vaccines, 53100 Siena, Italy

**Keywords:** Cytokine, Endotoxin, Lipopolysaccharide (LPS), Toll-like Receptor (TLR), Vaccine, GMMA, Outer Membrane Vesicles, Shigella, htrB, msbB

## Abstract

Outer membrane particles from Gram-negative bacteria are attractive vaccine candidates as they present surface antigens in their natural context. We previously developed a high yield production process for genetically derived particles, called generalized modules for membrane antigens (GMMA), from *Shigella*. As GMMA are derived from the outer membrane, they contain immunostimulatory components, especially lipopolysaccharide (LPS). We examined ways of reducing their reactogenicity by modifying lipid A, the endotoxic part of LPS, through deletion of late acyltransferase genes, *msbB* or *htrB*, in GMMA-producing *Shigella sonnei* and *Shigella flexneri* strains. GMMA with resulting penta-acylated lipid A from the *msbB* mutants showed a 600-fold reduced ability, and GMMA from the *S. sonnei* Δ*htrB* mutant showed a 60,000-fold reduced ability compared with GMMA with wild-type lipid A to stimulate human Toll-like receptor 4 (TLR4) in a reporter cell line. In human peripheral blood mononuclear cells, GMMA with penta-acylated lipid A showed a marked reduction in induction of inflammatory cytokines (*S. sonnei* Δ*htrB*, 800-fold; Δ*msbB* mutants, 300-fold). We found that the residual activity of these GMMA is largely due to non-lipid A-related TLR2 activation. In contrast, in the *S. flexneri* Δ*htrB* mutant, a compensatory lipid A palmitoleoylation resulted in GMMA with hexa-acylated lipid A with ∼10-fold higher activity to stimulate peripheral blood mononuclear cells than GMMA with penta-acylated lipid A, mostly due to retained TLR4 activity. Thus, for use as vaccines, GMMA will likely require lipid A penta-acylation. The results identify the relative contributions of TLR4 and TLR2 activation by GMMA, which need to be taken into consideration for GMMA vaccine development.

## Teaser

**Background:** GMMA from Gram-negative bacteria are an attractive vaccine technology, but lipopolysaccharide (LPS) reactogenicity limits use.

**Results:** Genetic LPS modification resulting in penta-acylation reduced *Shigella* GMMA reactogenicity to a TLR2-mediated limit. Modifications resulting in palmitoleoylated hexa-acylated LPS triggered higher TLR4-mediated reactogenicity.

**Conclusion:** Use of GMMA as vaccines will likely require LPS penta-acylation.

**Significance:** Understanding the relative contribution of TLR activation guides GMMA vaccine development.

## Introduction

Gram-negative bacteria naturally shed particles that consist of outer membrane lipids, outer membrane proteins, and soluble periplasmic components. These particles, called native outer membrane vesicles, have been proposed for use as vaccines (1). However, the yield is usually too low for a practical vaccine production. We have developed genetic modification of bacteria to induce high level shedding of particles called generalized modules for membrane antigens (GMMA)^2^ (2, 3) and the corresponding industrial processes required for a practical vaccine platform (2). In the case of *Shigella*, the required genetic modification is a deletion of the *tolR* gene whose corresponding protein is involved with linking the inner and outer membranes. This development is part of a program to develop an effective and affordable vaccine for the causative agents of shigellosis, a global human health problem, especially in developing countries and in children younger than 5 years (4), with more than 125 million cases (5) and 100,000 deaths per year (6). *Shigella* are Gram-negative bacteria divided into 50 different serotypes based on the carbohydrate composition of the O antigen of their lipopolysaccharide (LPS) (7). A limited number of serotypes contributes to the global burden of shigellosis, but the leading disease-causing serotypes vary between regions (8). The current globally dominant serotypes are *Shigella sonnei* and *Shigella flexneri* 2a, which account for more than 20% of shigellosis cases each (9, 10).

GMMA are highly immunogenic (2), in part, probably because of strong self-adjuvanticity. Because they are derived from the outer membrane of Gram-negative bacteria, they have high levels of LPS and lipoproteins, molecules that are strong activators of the innate immune response through recognition by different pattern recognition receptors, including Toll-like receptors (TLRs), a widely expressed sets of molecules present in mammalian cells (11). The receptors of particular importance for recognition of Gram-negative bacteria are TLR2 and TLR4. TLR2 is involved in the recognition of a wide range of pathogen-associated molecular patterns that include lipoproteins (di- or tri-acetylated) (11). TLR4 is the receptor involved in the recognition of LPS with MD-2, CD14, and LPS-binding protein (11, 12).

Depending on the dose of GMMA to be used in a vaccine, this strong activation of innate immunity may lead to unacceptable reactions in human subjects, *e.g.* a febrile response or, in extreme cases, septic shock (13), especially if parenterally administered. Further genetic manipulations of the bacteria could be used to reduce this risk. The single most abundant and highly potent immunostimulatory component in GMMA is LPS (1). Its structure is amenable to genetic modification to reduce the activation of the TLR4 pathway. To assess the effect of such genetic modifications, it is important to determine the relative contribution of TLR2 and TLR4 pathways to the reactogenicity of GMMA. This needs to be tested in human cells or cell lines containing the human TLR recognition system because the cytokine response induced varies considerably between mammalian species. For example, penta-acylated lipid A is a poor inducer of cytokines from human cells but is a strong stimulator of mouse cells (14, 15).

LPS consists of three main regions as follows: the glycolipid, lipid A, a core oligosaccharide, and an oligosaccharide chain (O antigen, OAg) usually consisting of 20–40 repeating units comprising 2–8 sugar molecules (16). Lipid A, which anchors the LPS to the outer membrane of bacteria, is the endotoxic part of LPS. The most endotoxic form of lipid A consists of a hexa-acylated glucosamine disaccharide phosphorylated at the 1 and 4′ position with acyl chains from 12 to 14 carbons in length and an asymmetric (4/2) distribution (14, 16, 17). This structure is common to *Escherichia coli* and *Shigella* (18, 19). During the synthesis of the lipid A, first a tetra-acylated core structure carrying *R*-3-hydroxymyristate at the 2-, 3-, 2′-, and 3′-positions, called lipid IVA, is generated, which is conserved in most of the Gram-negative bacteria (18, 19). Subsequently, the late acyltransferases HtrB (also called LpxL) and MsbB (also called LpxM) transfer a lauroyl fatty acid to the 3′-position (HtrB (20)) and a myristoyl fatty acid at position 2′ (MsbB (21)). In some bacteria there are alternative pathways leading to a modified structure of lipid A, and these may depend on environmental conditions, *e.g.* a palmitoleic acid is incorporated at the 3′-position (palmitoleoylation) instead of the lauroyl chain, catalyzed by LpxP in *E. coli* under cold shock conditions (22, 23).

The total number and the length of acyl chains plus the presence of the two phosphates in positions 1 and 4′ are critical factors for full lipid A activation of human TLR4/MD-2 (14, 16, 17, 19). Changes to the structure of the lipid A, either by removal of critical components or by replacement of one of the acyl chains by a different acyl residue, affects the binding and recognition by TLR4 and results in a lower endotoxicity *in vitro* (14, 16). A primary focus for reducing the reactogenicity of lipid A has been the modification of the acylation by inactivation of the genes encoding the late acyltransferases HtrB (20) and MsbB (21). The resulting predominantly penta-acylated lipid A showed a strong decrease of reactogenicity in human cells. For example, an *htrB* mutant of *Haemophilus influenzae* elicited a 40-fold lower expression of tumor necrosis factor-α (TNF-α) than the wild type (24), and purified LPS from an *E. coli* Δ*msbB* mutant had a 10,000-fold decreased ability to induce TNF-α production (25). *Shigella* carries two copies of the *msbB* gene, one encoded on the chromosome (*msbB1*) and the other on the virulence plasmid (*msbB2*) (26). Mutant strains of *S. flexneri* 5 lacking both copies showed an 11–20-fold lower capability to induce TNF-α from adherent human monocytes (26). An *msbB* double mutant strain of *S. flexneri* 2a was shown to elicit decreased cytokine responses from a murine macrophage line (27), but it has not been reported to be tested with human cells.

Previously, we showed that genetic lipid A modification is compatible with the high yield production process using a *S. sonnei* Δ*msbB* GMMA-producing strain (2). However, as that study focused on production, reactogenicity tests with the resulting GMMA were not performed. In this study, we investigate the effects of *msbB* and *htrB* deletions on the lipid A structure in GMMA-producing strains of *S. sonnei* and *S. flexneri* 2a and the relative activation by the resulting GMMA of the TLR4 and TLR2 pathways in human peripheral blood mononuclear cells (PBMC) and in cells transfected with human TLR4 and TLR2. We show that the penta-acylated lipid A generated by the *msbB* mutation has a 600-fold lower ability to stimulate TLR4 than wild-type lipid A, whereas the penta-acylated lipid A from the *S. sonnei* Δ*htrB* mutant is 60,000-fold less active. We further demonstrate that the cytokine production in PBMC stimulated by GMMA with penta-acylated lipid A is substantially reduced and similar for GMMA from *S. sonnei* Δ*htrB* (800-fold) and *S. sonnei* and *S. flexneri* 2a Δ*msbB* mutants (300-fold) and that the residual cytokine production is largely due to TLR2 activation. We also show that, in contrast to *S. sonnei* Δ*htrB*, a compensatory palmitoleoylation occurs in the *S. flexneri* 2a Δ*htrB* mutant resulting in a high proportion of hexa-acylated lipid A with substantial ability to activate TLR4, albeit requiring approximately an order of magnitude higher concentration than the isogenic cell line.

## EXPERIMENTAL PROCEDURES

### 

#### 

##### Strains and Generation of Mutations

*S. sonnei* 53G (28) and *S. flexneri* 2a 2457T (29) were chosen as parent strains. The list of *Shigella* mutant strains used in this study and their abbreviated identifications are listed in Table 1. As all strains used in this study are GMMA-producing strains, the abbreviated names only refer to their additional mutations and characteristics. For generation of mutants from *S. flexneri* 2a without virulence plasmid, a white colony was selected by white appearance on Congo red agar before the start of the genetic modification. The curing of the virulence plasmid (pINV) was confirmed by the absence of the origin of replication (*ori*) and the plasmid-encoded genes, *virG* and *ospD3*, using PCR. The primers are listed in Table 2. To generate the *tolR* deletion in *S. flexneri* 2a and plasmid-cured *S. flexneri* 2a-pINV, the same strategy and primers as described previously for the generation of the *S. sonnei* Δ*tolR* mutant (2) were used.

**TABLE 1:** **Strains used in this study and their abbreviations**

Strain name abbreviation	Genotype*^a^*	Modified lipid A	Ref.
*Ss*_−p − OAg_	*S. sonnei*–pSS Δ*tolR*::*kan*	−	2
*Ss*_−p − OAg_ Δ*msbB*	*S. sonnei*–pSS Δ*tolR*::*kan* Δ*msbB*::*cat*	+	2
*Ss*_−p − OAg_ Δ*htrB*	*S. sonnei*–pSS Δ*tolR*::*kan* Δ*htrB*::*cat*	+	This study
*Sf*2a_−p − OAg_	*S. flexneri* 2a–pINV Δ*tolR*::*kan* Δ*rfbG*::*erm*	−	This study
*Sf*2a_−p − OAg_ Δ*msbB*	*S. flexneri* 2a–pINV Δ*tolR*::*kan* Δ*rfbG*::*erm* Δ*msbB*::*cat*	+	This study
*Sf*2a_−p − OAg_ Δ*htrB*	*S. flexneri* 2a–pSS Δ*tolR*::*kan* Δ*rfbG*::*erm* Δ*htrB*::*cat*	+	This study
*Ss*_−p − OAg_ Δ*htrB* (pACYC*htrB*)	*S. sonnei*–pSS Δ*tolR*::*kat* Δ*htrB*::*cat* (pACYC*htrB*)	−	This study
*Sf*2a_−p − OAg_ Δ*htrB* (pACYC*htrB*)	*S. flexneri* 2a–pINV Δ*tolR*::*kan* Δ*rfbG*::*erm* Δ*htrB*::*cat* (pACYC*htrB*)	−	This study
*Sf*2a_+p − OAg_	*S. flexneri* 2a+pINV Δ*tolR*::*kan* Δ*rfbG*::*erm*	−	This study
*Sf*2a_+p + OAg_	*S. flexneri* 2a+pINV Δ*tolR*::*kan*	−	This study
*Sf*2a_−p + OAg_	*S. flexneri* 2a–pINV Δ*tolR*::*kan*	−	This study
*Sf*2a_+p + OAg_ Δ*htrB*	*S. flexneri* 2a+pINV Δ*tolR*::*kan* Δ*htrB*::*cat*	+	This study
*Sf*2a_−p + OAg_ Δ*htrB*	*S. flexneri* 2a–pINV Δ*tolR*::*kan* Δ*htrB*::*cat*	+	This study

*^a^* −pSS and −pINV, indicate that strain is cured of virulence plasmid; +pSS and +pINV, indicate that virulence plasmid is present.

**TABLE 2:** **Primers used in this study**

Primer name	Sequence 5′ → 3′
*htrB*-U1 Xba Sma	CTAGTCTAGAAACCCGGGCAATTGTATGTATTGTCG
*htrB-so*U2 SacI	ACTCGAGCTCCCGTCATCATCCAACGC
*htrB-flex*U2 SacI	ACTCGAGCTCATCCGATATACGTTCGCCC
*htrB-so*D1 SalI	ACGCGTCGACCTCAGTAATCAGGGTTCTTTG
*htrB-so*D2 SmaI	CTAACCCGGGTAAATCTCCCCTGCCGGATG
*htrB-flex*D1 SalI	ACGCGTCGACCCTGTAATCTCAGGTCAAATG
*htrB-flex*D2 SmaI	CTAACCCGGGTAAATCTCCCATGCCGGATG
*msbB-flex*U5 Sma	CTAGTCTAGAAACCCGGGTGATAGTGTAGCGGCACA
*msbB-flex*U3 Sac	ACTCGAGCTCGTGAGCAAAGCCAGCTG
*msbB-flex*D5 SalI	ACGCGTCGACCTCGGTGTGGAAATTGG
*msbB-flex*D3 Xba Sma	CTAACCCGGGCAACGTACTTACTCTACCG
*rfbF*-1stop Sma	CTAACCCGGGCTAAGCATCTAAGACACCATTCTGTATC
*rfbF*-2 SalI	ACGCGTCGACAATATCCTGGAGCATACGTGT
*rfc*-1 SacI	ACTCGAGCTCACCAATAACGCCTGTTTTCTG
*rfc*-2 XbaSma3	CTAGTCTAGAAACCCGGGCTTCTTTGTCGGCTTATTAGC
P1.*htrB*compl-EcoRI	ACCGGAATTCGTGTAACACTGGCATGGTGTA
P2.*htrB*compl-NcoI	CATGCCATTGTAGCAATCCGCTGTTGGTGCG
EcoRV.Ery.F	AGCTTGATATCAGAGTGTGTTGATAGTGCAGTATC
EcoRV.Ery.R	AGCTTGATATCACCTCTTTAGCTTCTTGGAAGCT
EcoRV.Cm.F	AGCTTGATATCTGTGACGGAAGATCACTTCG
EcoRV.Cm.R	AGCTTGATATCGGGCACCAATAACTGCCTTA
Ori-1	CGGCATCAGAATAATACAAGCAGC
Ori-2	AGGTGTACCGTGCTCTGGG
*virG*-1	GTCACAGGTAACATGACTCTGGAG
*virG*-2	CCATGTGTGAATACTACCTTCACCC
*ospD*3-1	GTTTTGCCTCATTCAAGATATCACC
*ospD*3-2	TGACGATGGTTTGTCAGGATTGC
*msbB*.F	CGCCAAAGTTCCGTGATCCCATT
*msbB*.R	CTCTTCGATGATCTCCAGCCCTT
*lpxP*.F	GGCTTTGGGTACAGCTTCCTTA
*lpxP*.R	CCAACCCTTCAACATCAAACC

The null mutation of *msbB1* (26), *htrB* (20), and *rfbG* (essential for OAg biosynthesis in *S. flexneri* 2a (30)) was obtained by replacing the gene of interest (“*gene*”) with an antibiotic resistance cassette, using the following strategy. The upstream and downstream regions of *gene* were amplified using the primer pairs *gene*-U and *gene*-D or *rfbF* and *rfc* (for the *rfG* knock out). The resistance cassette used to replace *gene* was amplified using primer pairs EcoRV.Ery.F/EcoRV.Ery.R or EcoRV.Cm.F/EcoRV.Cm.R. The fragments were inserted into pBluescript (Stratagene) so that the antibiotic resistance gene interposed the flanking regions of *gene*. The replacement construct (upstream region-resistance cassette-downstream region) was amplified using the primers binding to the 5′ end of the upstream flanking region and the 3′ end of the downstream flanking region of *gene* (see Table 2) and used to transform recombination-prone *tolR* deletion strains of *S. sonnei* or *S. flexneri* as described previously (2). In *S. sonnei*, the *htrB* gene was replaced by the chloramphenicol resistance gene *cat* (31). In *S. flexneri* 2a, *msbB1* and *htrB* were replaced by *cat,* and *rfbG* was replaced by the erythromycin resistance gene *erm* (32). In the *rfbG* knock out, also the flanking genes *rfbF* and *rfc* (30) were partially deleted. The *rfbG* deletion was introduced before the *msbB* or *htrB* deletion. The *msbB1* mutation was only introduced into the plasmid-cured strain as the plasmid carries a second copy of *msbB* (*msbB2* (26)). For simplicity, the mutant is referred to as Δ*msbB*.

To complement strains carrying the *htrB* deletion, the *htrB* gene was amplified from *S. sonnei* 53G, including 239 bp upstream and 172 bp downstream using primers P1.*htrB*compl-EcoRI and P2.*htrB*compl-NcoI and inserted into low copy vector pACYC184 (New England Biolabs). The resulting plasmid pACYC*htrB* was introduced into electrocompetent *S. sonnei* or S. *flexneri* 2a Δ*htrB* cells.

##### GMMA Production and Purification

Bacterial strains were routinely grown at 30 °C in liquid or on solid M9 medium supplemented with nicotinic acid (Na_2_HPO_4_ 7 g/liter, KH_2_PO_4_ 3 g/liter, NaCl 0.5 g/liter, NH_4_Cl 1 g/liter, 1 m MgSO_4_ 2 ml/liter, 1 m CaCl_2_ 0.1 ml/liter, glucose 0.4%, nicotinic acid 0.01 g/liter) or in chemically defined medium (SDM), with the same composition to the previously described SSDM (2) with the exception of the carbon source as follows: KH_2_PO_4_ 13.3 g/liter, (NH_4_)_2_HPO_4_ 4 g/liter, citric acid 1.7 g/liter, l-aspartic acid 2.5 g/liter, d-glucose 15 g/liter, CoCl_2_·6H_2_O 0.0025 g/liter, MnCl_2_·4H_2_O 0.015 g/liter, CuCl_2_·2H_2_O 0.0015 g/liter, H_3_BO_3_ 0.003 g/liter, Na_2_MoO_4_·2H_2_O 0.0025 g/liter, Zn(CH_3_COO)_2_·2H_2_O 0.0025 g/liter, ferric citrate 2 μm, MgSO_4_ 2 mm, thiamine 0.05 g/liter, nicotinic acid 0.01 g/liter, pH 6.7 (with NH_4_OH). When required, kanamycin (30 μg/ml), chloramphenicol (20 μg/ml), erythromycin (100 μg/ml), or tetracycline (20 μg/ml) were added.

For GMMA production, overnight cultures were grown in the presence of the specific selective antibiotics and used to inoculate the production medium to an OD of 0.03–0.05. Production cultures were incubated at 30 °C and 200 rpm overnight. Culture supernatants were collected by a 10-min centrifugation at 5,000 × *g* followed by a 0.22-μm filtration. GMMA were concentrated using an Amicon stirrer cell with a regenerated cellulose filter with a 100-kDa nominal molecular mass limit (Amicon Ultracell) under nitrogen flow. The retentate was collected in a 70-ml ultracentrifuge propylene tube (Beckman Coulter) and ultracentrifuged at 186,000 × *g* using 45Ti rotor (Beckman Coulter) for 2 h at 4 °C. Pellets were resuspended in 4 ml of PBS followed by 0.22-μm filtration. GMMA were stored at 4 °C.

##### GMMA Protein and KDO Quantification

GMMA quantities were expressed as total protein present in GMMA. The protein quantity was determined using the DC protein assay (Bio-Rad) kit (Lowry assay) according to the manufacturer's instructions. Bovine serum albumin (Pierce) was used for the standard curve in the range 1–50 μg/assay. Measurements of GMMA were performed in two different dilutions, each in duplicate.

Core-reducing end 2-keto-3-deoxyoctonate (KDO) after lipid A cleavage was quantified using the semicarbazide/HPLC-size exclusion chromatography method as we reported previously (33). To apply the method to GMMA, 150 μg of GMMA were hydrolyzed in 1% acetic acid for 3 h at 100 °C and subsequently centrifuged for 15 min at 14,000 × *g*. Supernatants were collected and dried in a SpeedVac, and the pellets were dissolved in water. Samples and a standard of 4–40 μg/ml KDO ammonium solution (Sigma, K2755) were derivatized using semicarbazide and analyzed by HPLC-size exclusion chromatography using a TSKgel G3000 PW-XL column (TOSOH, 808021). The amount of core reducing end KDO was calculated using the calibration curve built with the peak areas of derivatized KDO standard at 252 nm.

##### Negative Staining Transmission Electron Microscopy

A drop of 5 μl of GMMA suspension at a concentration of 100 μg/ml in PBS was adsorbed onto 300 mesh copper Formvar/carbon-coated grids for 5 min. Grids were then washed with a few drops of distilled water and dried by blotting with Whatman filter paper. For negative staining, grids were treated with 2% uranyl acetate in double distilled H_2_O for 1 min, blotted with Whatman filter paper, air-dried, and observed with a Tecnai 2 Spirit transmission electron microscope (FEI, Eindoven, The Netherlands) operating at 80 kV. Electron micrographs were recorded at a nominal magnification of 105,000×. GMMA diameters were measured manually on printed copies of the electron micrographs in comparison with the scale bar. The nonparametric Kruskal-Wallis test was used to statistically compare the sizes of GMMA obtained from different strains.

##### SDS-PAGE and Peptide Mass Fingerprinting

SDS-PAGE of GMMA was performed using 12% (w/v) polyacrylamide gels (Bio-Rad) as described previously (2). The gels were stained using Coomassie Blue stain (Sigma) according to the manufacturer's instructions for proteins.

Protein spots were excised from the gel and processed as described previously (34). Briefly, mass spectra were acquired on an Ultraflex MALDI TOF-TOF mass spectrometer (Bruker Daltonics) in reflectron, positive mode, in the mass range of 900 to 3,500 Da. Spectra were externally calibrated by using a combination of standards pre-spotted on the target (Bruker Daltonics). MS spectra were analyzed by peptide mass fingerprinting with FlexAnalysis (FlexAnalysis version 2.4, Bruker Daltonics). Peaks were annotated with FlexAnalysis default parameters and manually revised. Protein identification was carried from the generated peak list using the Mascot program (Mascot server version 2.2.01, Matrix Science). Mascot was run on a database containing protein sequences deduced from sequenced *Shigella* genomes (2).

##### MALDI-TOF and MS/MS Analysis on Lipid A

Lipid A was precipitated from GMMA using mild acid hydrolysis with 1% acetic acid for 2 h at 100 °C (35). Samples were centrifuged at 14,000 × *g* for 15 min; the pellets were resuspended in water and washed twice with water. The pellets were dried overnight using a SpeedVac and resuspended in chloroform/methanol 4:1 and mixed with an equal volume of Super DHB solution (Sigma). 2 μl of the mixture were loaded to the target plate (MTP 384 target plate ground steel BC, Bruker Daltonics) and analyzed by Ultraflex MALDI-TOF (Bruker Daltonics) in reflectron ion-negative mode. A peptide calibration standard (Bruker Daltonics), mixed with the Super DHB solution, was included in each analysis. For MS/MS analysis of lipid A, main peaks from the linear mode analysis were selected for collision-induced dissociation, and the resulting fragments were detected by MALDI TOF-TOF in ion negative mode. For each sample, spectra represent the integration of the analysis of 20 different areas of the spot by 50 single laser shots. The *m/z* rations were determined by Flex Analysis software in comparison with the peptide standard.

##### Quantitative Real Time PCR and RNA Isolation

RNA was purified using RNeasy Plus mini kit (Qiagen) from 2 ml of bacteria grown at 30 °C to an optical density of 1. Reverse transcription (2 μg of RNA/reaction) was performed using Superscript II, and the product was purified by QIAquick PCR purification kit (Qiagen). Quantitative RT-PCR for genes *msbB* and *lpxP* and was performed using 10 ng of cDNA, SYBR Green kit (Invitrogen), and 0.4 μm primers *msbB*.F/*msbB*.R and *lpxP*.F/*lpxP*.R in thermocycler MX3005P (Stratagene) with 40 cycles (95 °C, 15 s; 60 °C, 60 s). Fold induction was calculated as 2^−Δ(Δ*Ct*)^, were Δ*Ct* is the difference between the numbers of cycle of amplification needed to reach the threshold (fluorescence d*Rn* = 0.018) for *lpxP versus* the cycle of amplification of the late acyltransferase gene *msbB* of the constitutive lipid A biosynthesis pathway (36) in same strain. Δ(Δ*Ct*) represents the difference in the Δ*Ct* of *lpxP* in *Sf*2a_−p − OAg_ Δ*htrB* compared with *Ss*_−p − OAg_ Δ*htrB*.

##### NF-κB Luciferase Reporter Assay

TLR-specific activation assays were performed using human embryonic kidney 293 (HEK293) cells expressing luciferase under control of the NF-κB promoter and stably transfected with either TLR4, MD2, and CD14 (TLR4-HEK293) or TLR2 (TLR2-HEK293) (37). HEK293-transfected cells were maintained in DMEM complemented with 4.5 g/liter glucose and HEPES (Invitrogen), 10% fetal bovine serum (FBS), 1% penicillin/streptomycin solution (Invitrogen), and specific antibiotics for the different cell lines: puromycin (5 μg/ml), blasticidin (10 μg/ml), and hygromycin (250 μg/ml) for TLR4-HEK293 cells, and puromycin and hygromycin for TLR2-HEK293 cells.

For the NF-κB luciferase assay, 25,000 cells/well were seeded in 90 μl of complete DMEM without antibiotics in 96-well μClear® luciferase plates (PBI International) and incubated for 24 h at 37 °C. 10 μl of serial 5-fold dilutions of GMMA in PBS (0.0001–1,000 ng/ml final concentration in the assay) were added. All GMMA concentrations were tested in duplicate. After incubation for 5 h at 37 °C, supernatants were aspired from each well, and cells were lysed for 20 min at room temperature using 20 μl/well of 1:5 diluted “passive lysis buffer” (Promega). Produced luciferase was detected using 100 μl/well luciferase assay reagent (Promega), and emitted light was immediately quantified using a luminometer Lmax II^384^ (Molecular Devices). NF-κB activation of cells stimulated with GMMA is expressed as fold-increase of emitted light over the average result of PBS-stimulated control cells. GMMA concentrations needed to obtain a 3-fold (TLR4 experiments) or 10-fold (TLR2 experiments) induction of NF-κB were arbitrarily used for comparing relative activity because that was in the middle of the linear part of the sigmoidal curves. They were determined as *x* axis intercepts from the generated curves. The nonparametric Mann-Whitney test was used to statistically evaluate the results obtained with different GMMA.

##### PBMC Isolation, TLR Blocking, and Stimulation (Monocyte Activation Test)

Buffy coats from three different donors were used to isolate PMBC using Ficoll density centrifugation as reported previously (38). PBMC were cultured at a density of 2 × 10^5^ cells/well with 180 μl of RPMI 1640 medium complemented with 25 mm HEPES, 2 mm glutamine, 10% FBS, 1% penicillin/streptomycin (Invitrogen) in 96-well round bottom plates. After incubation at 37 °C for 30 min, 20 μl of 10-fold serial dilutions of GMMA in PBS (0.0001–1,000 ng/ml final concentration in the assay) were added. Each GMMA concentration was tested in duplicate. During experiment setup, PBMC were exposed to PBS as control to assess the baseline cytokine release and to determine the concentration of GMMA that did not trigger a significant activation of IL-6 release over baseline (0.0001 ng/ml). In further experiments, this GMMA concentration was used as background control. In blocking experiments, 25 μg/ml TLR4 blocking antibody (eBioscience) and/or 15 μg/ml TLR2 blocking antibody (eBioscience) in RPMI 1640 medium were added for 30 min before the addition of GMMA. Cells were incubated for 4 h at 37 °C, and supernatants were recovered after centrifugation of the plates at 400 × *g* and stored at −70 °C until analysis.

##### Cytokine Analysis by ELISA and 7-Plex Mesoscale

Nunc MaxiSorp 96-well plates were coated overnight at 4 °C with 2 μg/ml human IL-6 capture antibody (eBioscience 14-7069) in PBS, subsequently washed three times with PBS with 0.05% Tween 20 (PBST), blocked for 1 h with PBS with 1% BSA at room temperature, and washed three times with PBST. 50 μl of supernatants from PBMC experiments, diluted 1:4 with PBS, were incubated for 2 h at room temperature. A 2-fold dilution series of recombinant human IL-6 (eBioscience 39-8069) of 31.24 to 4,000 pg/ml in RPMI 1640 medium with 10% FBS was included as standard curve on each plate. Plates were washed three times with PBST. Bound IL-6 was detected using 2 μg/ml biotin-conjugated anti-human IL-6 (eBioscience 13-7068) in PBST with 0.1% BSA for 2 h at room temperature, followed by three washes with PBST, 20 min of incubation at room temperature with streptavidin/horseradish peroxidase (R&D Systems, DY998) diluted 1:200 in PBST with 0.1% BSA, three washes with PBST, and a color reaction with 100 μl/well substrate (R&D Systems, DY999) for 8 min at room temperature in the dark. The reaction was stopped by adding 50 μl/well of 12.5% sulfuric acid. The plates were read at 450 and 630 nm and the A_450–630 nm_ was determined. IL-6 concentrations in the samples were calculated in comparison with the standard. Results below the detection limit were assigned half of the detection limit.

Mesoscale 7-spot (MSD Technology) analysis for cytokines IL-6, IL-8, IL-1β, TNF-α, IL-10, IL-12, and IFN-γ was performed with 25 μl of supernatants from PBMC according to the manufacturer's instructions. Concentrations of the different cytokines in the samples were determined in comparison with the preloaded standard in the plates.

For the analysis of the cytokine release by PBMC, the average cytokine levels in the duplicate assays were plotted against the GMMA concentration. GMMA concentrations needed to obtain a 10-fold increase of IL-6 release over the average level obtained at the lowest GMMA concentration (background) were determined as *x* axis intercepts from the generated curves and used to compare the stimulatory activity of the different GMMA. The nonparametric Mann-Whitney test was used to statistically evaluate the results obtained with different GMMA.

For statistical analysis of the results of the TLR blocking experiments, the ratio of IL-6 produced by PBMC treated with anti-TLR4 or anti-TLR2 and the IL-6 produced by PBMC not treated with blocking antibodies, stimulated with the same concentration of GMMA, were calculated to normalize between the different experiments using PBMC from different donors. The ratio was determined for each replicate in the experiments. The nonparametric Wilcoxon signed rank test was used to assess whether the obtained ratios were significantly different from 1 (no effect by blocking).

## RESULTS

### 

#### 

##### Mutant Production and Conditions for GMMA Production

We previously reported the generation of a *S. sonnei-*pSS Δ*tolR* Δ*msbB* mutant (*Ss*_−p − OAg_ Δ*msbB*) that was able to grown to high optical densities in chemically defined medium developed for fermentation at 30 °C. The *Ss*_−p − OAg_ Δ*msbB* strain was cured of the virulence plasmid (2) to remove a second copy of the *msbB* gene (*msbB2* (26)) and the OAg biosynthesis genes (39) encoded on the plasmid. Thus, we chose a plasmid-deficient background to generate the *htrB* mutant of *S. sonnei* (*Ss*_−p − OAg_ Δ*htrB*) and also used a plasmid- and OAg-deficient background in *S. flexneri* 2a to compare GMMA from the respective Δ*msbB* and Δ*htrB* mutants to the GMMA from the *S. sonnei* strains.

Similar to the *Ss*_−p − OAg_ Δ*msbB* mutant (2), the *Sf*2a_−p − OAg_ Δ*msbB* mutant retained the ability to grow in complex media at 37 °C but at a slower rate (2-fold duplication time) than a single Δ*tolR* mutant. In contrast, Δ*htrB* strains only grew in chemically defined or minimal media and only at 30 °C. The duplication time of the Δ*htrB* or Δ*msbB* strains increased from 30 min to ∼2 h, but all the strains were able to reach high ODs (OD 10) after overnight incubation in chemically defined medium at 30 °C. Thus 30 °C and chemically defined medium were chosen as the standard growth conditions. All strains with lipid A modifications yielded more than 50 mg/liter GMMA protein.

**FIGURE 1.**
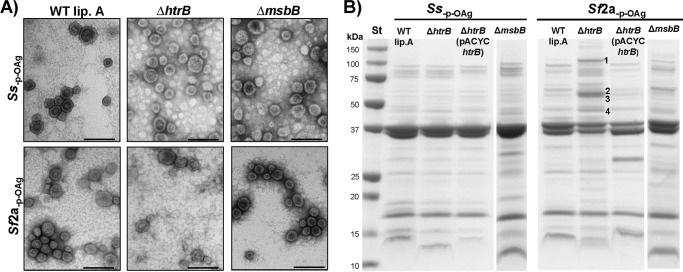
*A,* electron microscopy of GMMA from different strains. GMMA were purified from *Ss*_−p − OAg_ and *Sf*2a_−p − OAg_ containing wild-type lipid A (*Ss*_−p − OAg_ *WT lip. A, Sf*2a_−p − OAg_ *WT lip. A*), *Ss*_−p − OAg_ Δ*msbB*, *Sf*2a_−p − OAg_ Δ*msbB*, *Ss*_−p − OAg_ Δ*htrB*, and *Sf*2a_−p − OAg_ Δ*htrB*, negatively stained, and viewed by electron microscopy (105,000-fold magnification) revealing the presence of well organized membrane particles with diameters ranging between 17 and 53 nm in each preparation. *Bar length,* 100 nm. *B,* SDS-PAGE. 10 μg (protein) of the GMMA shown in *A* and GMMA from *Ss*_−p − OAg_ Δ*htrB* (pACYC*htrB*) and *Sf*2a_−p − OAg_ Δ*htrB* (pACYC*htrB*) were separated by SDS-PAGE (12% polyacrylamide) and Coomassie stained. Four protein bands that were more abundant in GMMA from *Sf*2a_−p − OAg_ Δ*htrB* than in GMMA from other *Sf*2a_−p − OAg_ strains were identified by peptide mass fingerprinting: *1,* pyruvate dehydrogenase; *2,* glutamine synthetase; *3,* ketol-acid reductoisomerase; *4,* d-3-phosphoglycerate dehydrogenase.

GMMA from *Shigella* strains carrying different mutations showed similar morphology by electron microscopy (Fig. 1*A*) with average sizes of 30–32 nm in all six strains and a size distribution of 17–53 nm, measured with 30 GMMA per strain. A comparison of the GMMA sizes from all strains gave no significant difference (*p* = 0.90). To characterize whether the genetic lipid A modifications might alter the protein composition of GMMA, the protein pattern of GMMA from the different mutants was evaluated by SDS-PAGE (Fig. 1*B*). Although the overall pattern remained similar, four protein bands, identified as pyruvate dehydrogenase, glutamine synthetase, ketol-acid recutoisomerase, and d-3-phosphoglycerate dehydrogenase (Fig. 1*B*) by peptide mass fingerprinting, were found to be up-regulated in GMMA from *Sf*2a_−p − OAg_ Δ*htrB*. As these proteins are cytoplasmic proteins, no effect on the reactogenicity studies was expected.

##### Characterization of Lipid A by MALDI-TOF and MALDI-TOF/TOF

**FIGURE 2.**
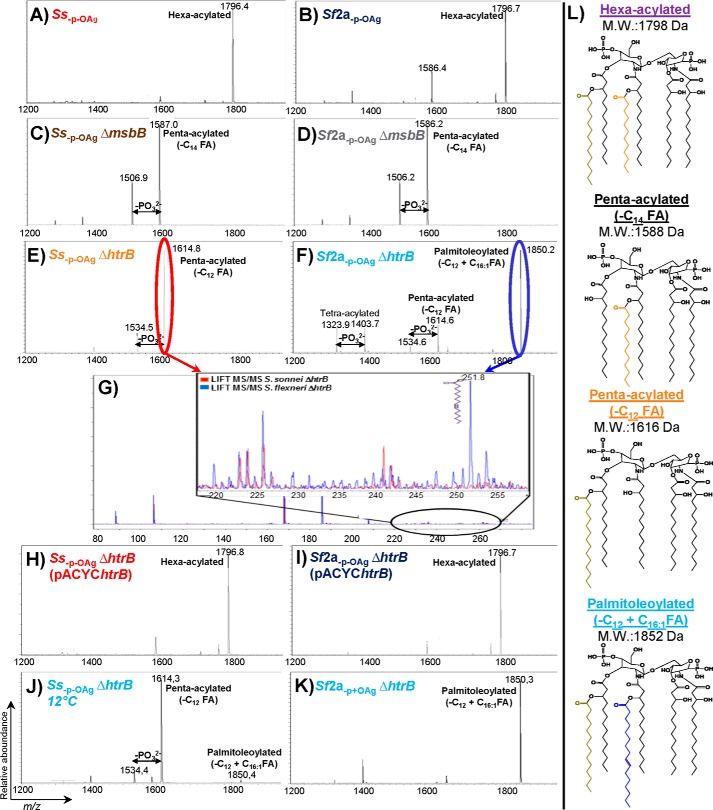
**MALDI-TOF spectra of lipid A preparations in reflectron ion-negative mode.** Lipid A was extracted from GMMA from the following: *A, Ss*_−p − OAg_; *B, Sf*2a_−p − OAg_; *C, Ss*_−p − OAg_ Δ*msbB*; *D, Sf*2a_−p − OAg_ Δ*msbB*; *E, Ss*_−p − OAg_ Δ*htrB*; *F, Sf*2a_−p + OAg_ Δ*htrB; H, Ss*_−p − OAg_ Δ*htrB* (pACYC*htrB*); *I, Sf*2a_−p − OAg_ Δ*htrB* (pACYC*htrB*); *J, Ss*_−p − OAg_ Δ*htrB* grown at 12 °C; and *K, Sf*2a_−p + OAg_ Δ*htrB. G,* overlay of negative ion LIFT MALDI-TOF/TOF spectra in the low *m/z* range of the dominant species in lipid A from *Ss*_−p − OAg_ Δ*htrB* (*E*) and *Sf*2a_−p − OAg_ Δ*htrB* (*F*) after collision-induced dissociation. *L,* lipid A structures with molecular weights corresponding to the observed main peaks.

The lipid A of LPS of the mutants was extracted and analyzed by MALDI-TOF. The spectra are reported in Fig. 2, and the structures of lipid A corresponding to the main peaks were assigned on the basis of mass and by comparison of results with similar mutants of *E. coli* (Fig. 2*L*) (40). The main peaks in the mass spectra obtained by MALDI-TOF from lipid A purified from GMMA from *S. sonnei* and *S. flexneri* 2a strains with wild-type (WT) LPS (Fig. 2, *Ss*_−p − OAg_ (*A*) and *Sf*2a_−p − OAg_ (*B*)) had an *m/z* corresponding to the theoretical mass of the hexa-acylated lipid A of 1,798 Da. The main peaks obtained by mass spectrometry from the *Ss*_−p − OAg_ Δ*msbB* GMMA (Fig. 2*C*) and *Sf*2a_−p − OAg_ Δ*msbB* (Fig. 2*D*) GMMA corresponded, in both strains, to a penta-acylated lipid A lacking a myristoyl chain (theoretical mass 1,588 Da, 210 *m/z* shift to WT lipid A due to the absence of a C_14_ fatty acid chain) consistent with *msbB* knock outs.

The mass spectrum of lipid A from *Ss*_−p − OAg_ Δ*htrB* GMMA (Fig. 2*E*) showed a main peak corresponding to a penta-acylated lipid A lacking a lauroyl chain (theoretical mass 1,616 Da, corresponding to the absence of a C_12_ fatty acid chain giving a *m/z* shift of 182), consistent with an *htrB* knock out. The spectra obtained from GMMA of *Sf*2a_−p − OAg_ Δ*htrB* (Fig. 2*F*) also had the penta-acylated lipid A species with the deletion of a lauroyl chain but showed a new peak at *m/z* 1,850 (Fig. 2*F*), most likely corresponding to a hexa-acylated lipid A species with an *m/z* different from WT lipid A. The mass of this lipid A species corresponds to acylation by a palmitoleoyl chain (a C_16:1_ fatty acid chain, *m/z* shift of 236) of the penta-acylated lipid A (*m/z* 1,616) present in both the *Ss*_−p − OAg_ Δ*htrB* GMMA and of *Sf*2a_−p − OAg_ Δ*htrB* to give a hexa-acylated lipid A. Confirmation for the palmitoleoylation was obtained by MS/MS analysis using collision-induced decay to fragment the lipid A species present in the main peaks of the first dimension MS for GMMA from *Ss*_−p − OAg_ Δ*htrB* (*m/z* 1,615, Fig. 2*E*) and *Sf*2a_−p − OAg_ Δ*htrB* (*m/z* 1,850, Fig. 2*F*). The main difference observed when overlaying the MS/MS spectra was a peak with an *m/z* corresponding to a palmitoleoyl chain (*m/z* 252, highlighted in Fig. 2*G*) in *Sf*2a_−p − OAg_ Δ*htrB*. MALDI-TOF spectra of GMMA from the *Ss*_−p − OAg_ Δ*htrB* and *Sf*2a_−p − OAg_ Δ*htrB* strains complemented with pACYC*htrB* (Fig. 2, *H* and *I*) showed in both cases a hexa-acylated WT lipid A as the main peak (observed, *m/z* 1,797; theoretical, *m/z* 1,797), and no hepta-acylated peak (theoretical *m/z* 2,034 for a wild-type with extra palmitoleoyl chain) was observed.

The palmitoleoylated hexa-acylated form (*m/z* 1,852) was also the main form in MALDI-TOF spectra of lipid A purified from GMMA from *Sf*2a_−p + OAg_ (Fig. 2*K*), *Sf*2a_+p + OAg_, and *S. flexneri* 3a and 6 ΔtolR Δ*htrB* strains.^3^

To test whether the production of the palmitoleoylated hexa-acylated lipid A species (*m/z* 1,851) could be induced in *S. sonnei* under stress conditions, *Ss*_−p − OAg_ Δ*htrB* was grown at 12 °C to induce a cold stress response. In the corresponding lipid A analysis by MALDI-TOF, a small amount (signal intensity less than 5% of the main species) of the palmitoleoylated lipid A species was identified (Fig. 2*J*).

Palmitoleoylation of lipid A in the absence of the lauroyl chain is consistent with the activity by the late acyltransferase LpxP. Thus, the expression level of *lpxP* was quantified in *Ss*_−p − OAg_ Δ*htrB* and *Sf*2a_−p − OAg_ Δ*htrB* grown at 30 °C in comparison with the *msbB* gene that is part of the constitutive lipid A pathway using real time PCR. In three independent experiments, the ratio of transcript of *lpxP* to *msbB* was on average 7.3-fold higher (standard deviation 0.9) in *Sf*2a_−p − OAg_ Δ*htrB* compared with *Ss*_−p − OAg_ Δ*htrB*.

##### TLR4-NF-κB Luciferase Reporter Assay

**FIGURE 3.**
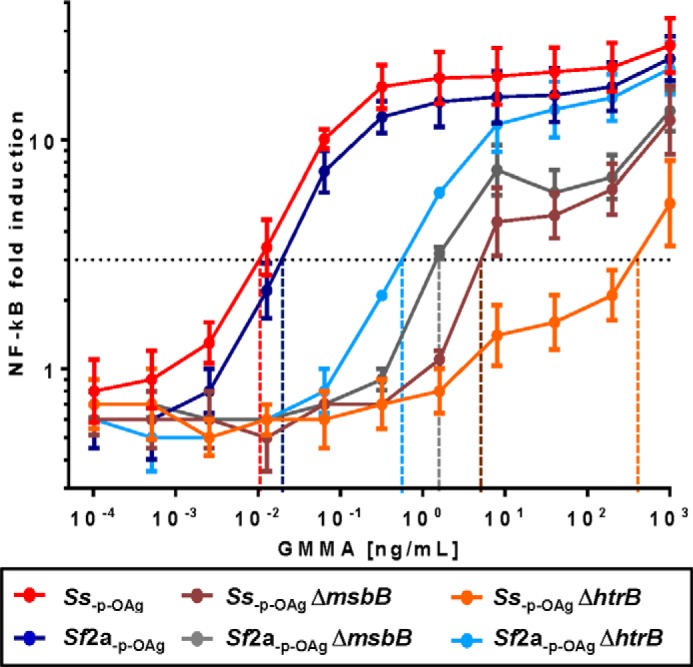
**Activation of TLR4 reporter cell line by different GMMA.** 25,000 TLR4-HEK293 cells/well were stimulated with 0.0001–1000 ng/ml (5-fold steps) of GMMA obtained from *Ss*_−p − OAg_, *Sf*2a_−p − OAg_, *Ss*_−p − OAg_ Δ*msbB*, *Sf*2a_−p − OAg_ Δ*msbB*, *Ss*_−p − OAg_ Δ*htrB*, and *Sf*2a_−p − OAg_ Δ*htrB*. After 5 h, luciferase expression was measured and expressed as fold-induction compared with cells incubated with PBS and plotted as averages of duplicates with standard deviations. GMMA concentrations that resulted in 3-fold increased activation of NF-κB (*black dashed line*) over background are shown as *x* axis intercepts (*colored dashed lines*). A representative result of four independent experiments is shown.

We stimulated HEK293 cells stably transfected to express only human TLR4 recognition complex and an NF-κB-inducible luciferase reporter gene (41) to characterize TLR4 stimulation by GMMA with the different lipid A species. The results obtained with different concentrations of GMMA are displayed in Fig. 3 and Table 3.

**TABLE 3:**
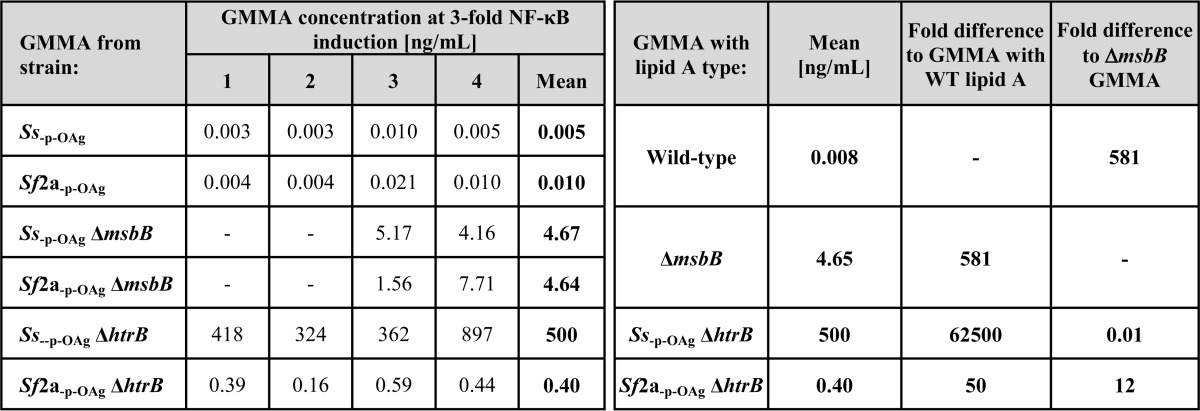
**TLR4 NF-κB-specific assay results** Concentrations of each type of GMMA resulting in 3-fold increased activation of NF-κB were determined in four independent experiments (wild-type and Δ*htrB* GMMA) or two independent experiments (Δ*msbB* GMMA) using TLR4/MD2/CD14 HEK293 transfectant cells as shown in Fig. 3. Results from GMMA from *Ss*_−p − OAg_ and *Sf*2a_−p − OAg_ with wild-type lipid A and GMMA from *Ss*_−p − OAg_ Δ*msbB* and *Sf*2a_−p − OAg_ Δ*msbB* (Δ*msbB* GMMA) were combined for further analysis. Fold differences of the mean concentration of the respective GMMA to stimulate 3-fold NF-κB induction were calculated in comparison with GMMA with wild-type lipid A and Δ*msbB* GMMA.

GMMA from *Sf*2a_−p − OAg_ Δ*msbB* and *Ss*_−p − OAg_ Δ*msbB* stimulated similar levels of NF-κB expression in the HEK293 TLR4 transfectant cells (Fig. 3) and required ∼600-fold more GMMA than the parent GMMA (Table 3) to give a 3-fold increase in NF-κB activity. In contrast, GMMA from *Ss*_−p − OAg_ Δ*htrB* and *Sf2a*_−p − OAg_ Δ*htrB* gave very different results. NF-κB induction by *Ss*_−p − OAg_ Δ*htrB* GMMA was only detectable at a high concentration, requiring ∼60,000-fold more GMMA than the GMMA with wild-type lipid A and 100-fold more than Δ*msbB* GMMA to stimulate the same level of NF-κB activity (Fig. 3 and Table 3). In contrast, *Sf*2a_−p − OAg_ Δ*htrB* GMMA (Fig. 3 and Table 3) retained higher TLR4 stimulation and required 10-fold less GMMA (*p* = 0.0286) than Δ*msbB* GMMA to result in a similar induction of NF-κB. Accordingly, the decrease of TLR4 stimulation compared with GMMA with wild-type lipid A was the smallest (50-fold) of all tested GMMA with lipid A modifications.

To ensure that the observed differences are the results of differences of the stimulatory activity of the lipid A and not caused by different amounts of lipid A present in GMMA, the molar amount of lipid A per mg of protein was determined by quantifying the LPS core sugar KDO. The amounts of lipid A were similar in all GMMA. In comparison with GMMA from *Ss*_−p − OAg_ Δ*htrB* with the lowest activity, GMMA from *Ss*_−p − OAg_ contained 1.1-fold, *Sf*2a_−p − OAg_ 2.0-fold, *Sf*2a_−p − OAg_ Δ*htrB* 0.3-fold, *Ss*_−p − OAg_ Δ*msbB* 0.8-fold, and *Sf*2a_−p − OAg_ Δ*msbB* 1.1-fold the amount of lipid A.

##### Cytokine Release from Human PBMC

**FIGURE 4.**
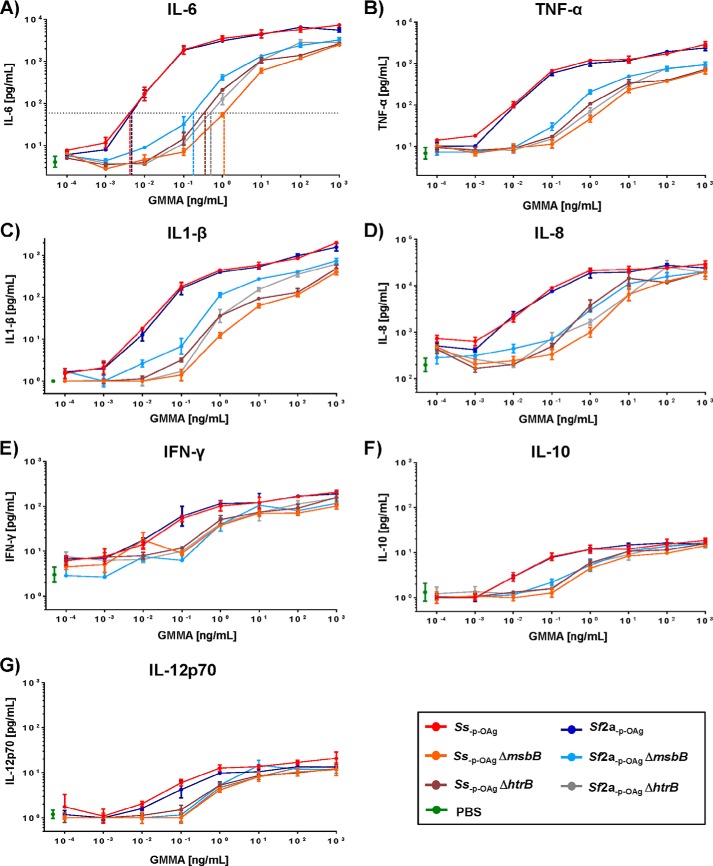
**Cytokine release by human PBMC after stimulation with different types of GMMA.** 200,000 human PBMC cells were stimulated for 4 h with 0.0001–1000 ng/ml (10-fold steps) of GMMA from *Ss*_−p − OAg_, *Sf*2a_−p − OAg_, *Ss*_−p − OAg_ Δ*msbB*, *Sf*2a_−p − OAg_ Δ*msbB, Ss*_−p − OAg_ Δ*htrB*, and *Sf*2a_−p − OAg_ Δ*htrB*. Release of the following: *A,* IL-6; *B,* TNF-α; *C,* IL-1β; *D,* IL-8; *E,* interferon-γ (IFN-γ); *F,* IL-10; and *G,* IL-12 p70, were measured by human pro-inflammatory 7-plex mesoscale and plotted as averages of duplicates with standard deviations. GMMA concentrations that resulted in 10-fold increase of IL-6 release over background are shown as *x* axis intercepts (*colored dashed lines*). Cytokine release by PBMC exposed to PBS was used as control.

In order measure the endotoxin level of GMMA in a more natural context, and in particular to examine whether the GMMA could stimulate additional pattern recognition receptors to TLR4, GMMA purified from different mutants were used to stimulate human PBMC in the monocyte activation test (Fig. 4). GMMA purified from *Shigella* strains without lipid A modification (both *Ss*_−p − OAg_ and *Sf*2a_−p − OAg_) induced high levels of the pro-inflammatory cytokines interleukin 6 (IL-6), TNF-α, IL-1β, and IL-8, intermediate levels of IFN-γ, and low levels of IL-12 and IL-10 (Fig. 4). All GMMA with lipid A modifications resulted in substantially lower cytokine release (Fig. 4).

The GMMA concentration required to give a 10-fold increase in IL-6 release over background (Table 4) was used for comparing relative activity (42). The same analysis performed at other points within the linear part of the curve (3- and 30-fold over background, respectively) gave similar results (data not shown).

**TABLE 4:**
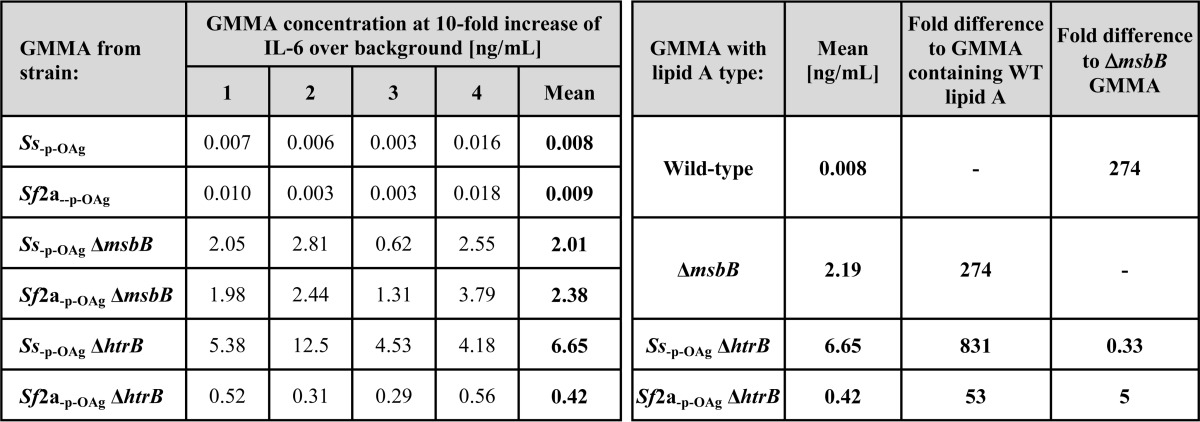
**Monocyte activation test results from different blood donors** Concentrations of each type of GMMA resulting in 10-fold increased release of IL-6 were determined using PMBC from four different donors as shown in Fig. 4. Results for GMMA from *Ss*_−p − OAg_ and *Sf*2a_−p − OAg_ with wild-type lipid A and GMMA from *Ss*_−p − OAg_ Δ*msbB* and *Sf*2a_−p − OAg_ Δ*msbB* (Δ*msbB* GMMA) were compared by the Mann-Whitney test (*p* = 0.8824 and *p* = 1.000, respectively) and combined for further analysis. Fold differences of the mean concentration of the respective GMMA to stimulate a 10-fold increase of IL-6 release were calculated in comparison of GMMA with wild-type lipid A and with Δ*msbB* GMMA.

GMMA purified from *Shigella* strains carrying lipid A modification gave a similar rank order in reduction of IL-6 release upon stimulation in comparison with the parent strains with wild-type lipid A (Fig. 4 and Table 4), as observed in the TLR4-specific assay (Fig. 3 and Table 3) but with smaller differences than in the TLR4-specific assay. The amount of GMMA required to give a 10-fold increase in IL-6 release was as follows: *Ss*_−p − OAg_ Δ*htrB* (800×) > *Ss*_−p − OAg_ Δ*msbB* ≈ *Sf*2a_−p − OAg_ Δ*msbB* (300×) > *Sf*2a_−p − OAg_ Δ*htrB* (50×) the amount of GMMA wild-type lipid A (Table 4).

##### TLR Blocking

**FIGURE 5.**
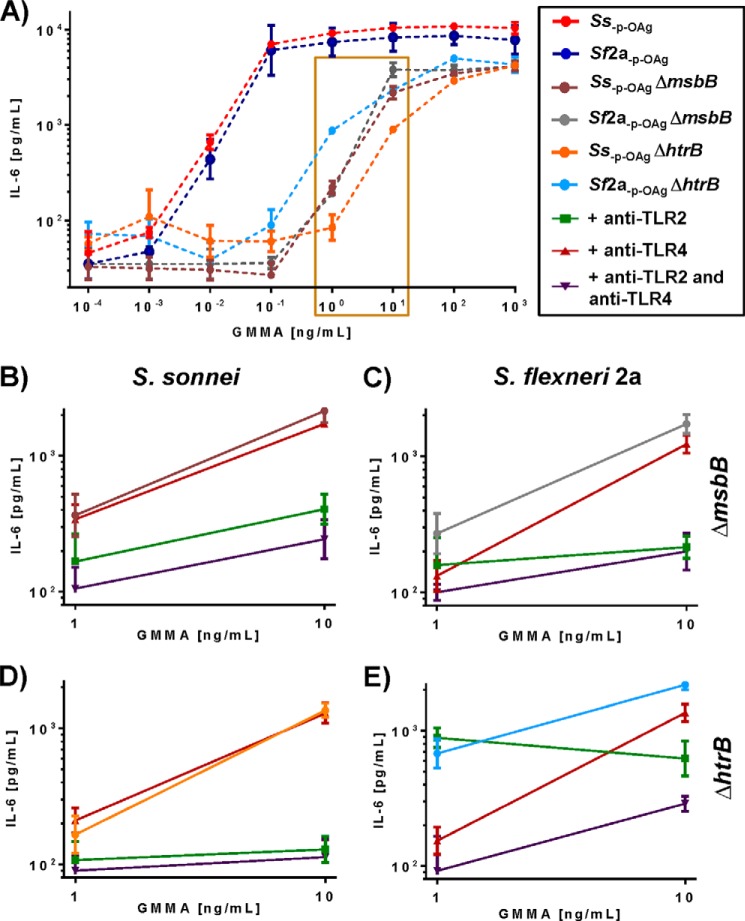
**TLR blocking experiments.** 200,000 human PBMC cells were stimulated with GMMA from different strains. After a 4-h incubation, IL-6 release was measured by ELISA and plotted as average of duplicates with standard deviation. *A,* IL-6 release after stimulation with 0.0001–1000 ng/ml (10-fold steps) of GMMA from different strains. The *rectangle* highlights the concentration of GMMA used in blocking experiments. *B–E,* cells were incubated with 25 μg/ml anti-TLR4 (*dark red graphs*), 15 μg/ml anti-TLR2 (*green graphs*), or both (*violet graphs*) for 30 min before exposure to 1 or 10 ng of GMMA from *Ss*_−p − OAg_ Δ*msbB* (*B*); *Sf*2a_−p − OAg_ Δ*msbB* (*C*); *Ss*_−p − OAg_ Δ*htrB* (*D*); and *Sf*2a_−p − OAg_ Δ*htrB* (*E*). The graphs from experiments without blocking are shown in the same color as in *A*. A representative result of three independent experiments is shown.

With the objective of identifying the TLRs that contribute to the residual activation by GMMA with lipid A modification, PBMC were incubated with TLR2 and/or TLR4 blocking antibodies before stimulation with 1 and 10 ng/ml of GMMA, concentrations chosen to give a significant but not saturating increase of IL-6 (Fig. 5). The three GMMA with penta-acylated lipid A, *Ss*_−p − OAg_ Δ*htrB* (Fig. 5*D*), *Ss*_−p − OAg_ Δ*msbB* (Fig. 5*B*), and *Sf*2a_−p − OAg_ Δ*msbB* (Fig. 5*C*), gave similar results as follows: IL-6 production was substantially reduced following incubation with TLR2 blocking antibody (70–90%) but either no reduction or minimal reduction was observed with TLR4 blocking antibody (10–30%) suggesting residual activity was principally due to TLR2 activation. With the *Ss*_−p − OAg_ Δ*msbB* and *Sf*_−p − OAg_ Δ*msbB* GMMA, a small reduction was obtained with the TLR4 blocking antibody alone (*Ss*_−p − OAg_ Δ*msbB*, *p* = 0.0234; *Sf*2a_−p − OAg_ Δ*msbB*, *p* = 0.0078 in four experiments) suggesting that there was still residual TLR4 activation. Further reduction was achieved with the combination of TLR2 and TLR4 blocking antibodies compared with TLR2 blocking antibody alone. With the *Ss*_−p − OAg_Δ*htrB* GMMA, no effect from TLR4 blocking was observed.

The GMMA with mostly hexa-acylated lipid A (*Sf*2a_−p − OAg_ Δ*htrB*, Fig. 5*E*) gave a substantial reduction with a TLR4 blocking antibody, *i.e.* 80% reduction at 1 ng/ml and 40% at 10 ng/ml GMMA concentration. TLR2 blocking showed no effect on the IL-6 release at 1 ng/ml GMMA concentration and resulted in ∼35% reduction (average of three independent experiments) at the 10 ng/ml GMMA concentration. Incubation with both TLR2 and TLR4 blocking antibodies gave lower IL-6 production at 1 and 10 ng/ml GMMA suggesting that both TLR were still active but that the TLR4 activation was dominant especially at lower GMMA concentrations.

**FIGURE 6.**
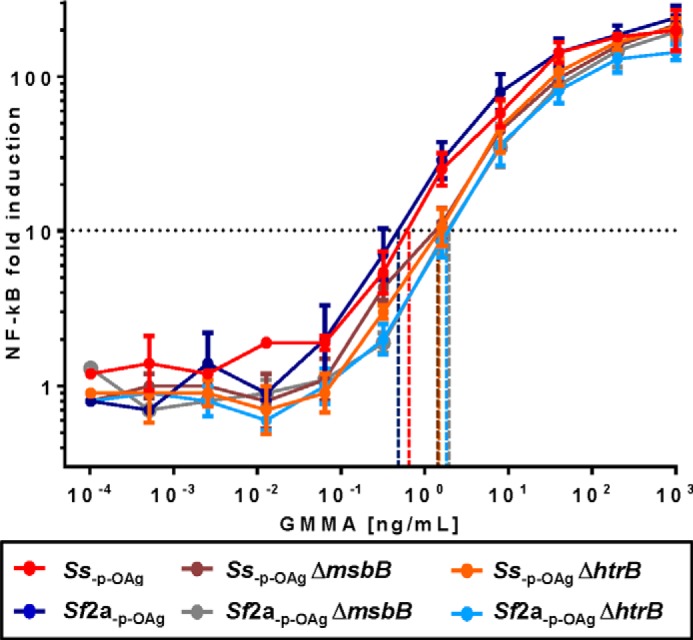
**Activation of TLR2 reporter cell line by different GMMA.** 25,000 TLR2-HEK293 cells/well were stimulated with 0.0001–1000 ng/ml (5-fold steps) of GMMA from *Ss*_−p − OAg_, *Sf*2a_−p − OAg_, *Ss*_−p − OAg_ Δ*msbB*, *Sf*2a_−p − OAg_ Δ*msbB, Ss*_−p − OAg_ Δ*htrB*, and *Sf*2a_−p − OAg_ Δ*htrB*. After 5 h, luciferase expression was measured and expressed as fold-induction compared with cells incubated with PBS. Average induction levels and standard deviations of duplicates are plotted. The GMMA concentrations that resulted in 10-fold increased activation of NF-κB (*black dashed line*) over the average induction at the lowest concentration of GMMA are shown as *x* axis intercepts (*colored dashed lines*). A representative result of four independent experiments is shown.

To confirm that the differences in the relative contribution of TLR4 and TLR2 to activation observed in the blocking experiments were primarily dependent on the differential TLR4 activation by the different GMMA, the ability of the GMMA to activate TLR2 was tested by stimulating HEK293-TLR2 transfectant cells. All four Δ*msbB* or Δ*htrB* GMMA required similar GMMA concentrations (2.6–4.9 ng/ml) to give a 10-fold NF-κB induction (Fig. 6).

## DISCUSSION

GMMA are attractive candidates for vaccines as they present surface antigens in their natural environment and conformation. For use as vaccines, dependent on the dose, the reactogenicity needs to be reduced, as GMMA contain LPS and other TLR stimulatory components, *e.g.* lipoproteins. Previously, we described a high yield production process for GMMA (2). The goal of this study was to demonstrate the impact of reducing the endotoxin potential of *Shigella* GMMA by genetic modification of the lipid A component of the GMMA LPS.

The results of deleting the *msbB* gene from both *S. sonnei* Δ*tolR* and *S. flexneri* 2a Δ*tolR* and by deleting the *htrB* gene from *S. sonnei* Δ*tolR* were as expected: conversion of a hexa-acylated lipid A to a penta-acylated lipid A through loss of a myristic acid (Δ*msbB*) or lauric acid (Δ*htrB*). However, we have demonstrated the presence of a palmitoleoyl chain in lipid A purified from *Sf*_−p − OAg_ Δ*htrB* GMMA.

Palmitoleoylation in *lpxL* (*htrB*) mutants has previously been reported in *E. coli*. It was shown to be a moderately abundant species at 30 °C and as the dominant species at 37 °C (43). In *S. flexneri* 2a Δ*htrB*, the signal of palmitoleoylated lipid A was the dominant species at 30 °C, with very little signal due to the penta-acylated species present. Similar results were obtained for the *S. flexneri* 3 and *S. flexneri* 6 strains tested.

The inability to detect a hepta-acetylated lipid A in either the *Sf*2a_−p − OAg_ GMMA or the *Sf*2a_−p − OAg_ Δ*htrB* GMMA complemented with *htrB* expression from pACYC*htrB* suggests that the palmitoleoylation is on the same site occupied by lauroyl acid in the wild-type lipid A, although further studies would be required to prove this. This would also be consistent with palmitoleoylation catalyzed by the action of LpxP, a late acyltransferase that acts at the HtrB site, as part of what has been described as a cold response in *E. coli* (23). Thus, the palmitoleoylation could be part of a stress response. Similarly, in the *E. coli* Δ*lpxL* mutant, the abundance of the palmitoleoylated lipid A increased with stress (growth at 37 °C (43)). In Δ*tolR* mutant strains, the *tolR* mutation could provide stress and thus result in higher abundance of palmitoleoylated lipid A at 30 °C, although why this was not found in the *Ss*_−p − OAg_ Δ*htrB* strain examined is not clear. We were able to observe a small signal due to palmitoleoylated lipid A in this *S. sonnei* strain grown at 12 °C indicating that there was a functional LpxP present. However, using quantitative real time PCR, we found a 7-fold higher gene expression of *lpxP* in *Sf*2a_−p − OAg_ Δ*htrB* compared with in *Ss*_−p − OAg_ Δ*htrB* grown at 30 °C, possibly resulting in a higher level of LpxP protein present at 30 °C in the *S. flexneri* 2a Δ*htrB* strain.

All Δ*htrB S. flexneri* isolates examined had the palmitoleoylation of the lipid A regardless of the presence or type of OAg on the LPS (*S. flexneri* 2a, 3, and 6 have different O antigens) or the presence of the virulence plasmid (*Sf*2a_−p + OAg_ Δ*htrB*, *Sf*2a_+p + OAg_ Δ*htrB*). For these, palmitoleoylation might be a strongly selected compensation after the *htrB* knock out. The reason for this is unclear. However, in line with this hypothesis, all of our attempts to introduce an *lpxP* knock out into *S. flexneri* 2a Δ*htrB* failed.

Although the impact of lipid A modifications on TLR4 activation has been widely studied (24–27, 44), the availability of GMMA from isogenic *Shigella* lines, all with similar concentrations of lipid A, enabled us to compare in detail the residual ability of modified lipid A to activate human TLR4 receptors either in HEK293-TLR4 transfectant cells or in human PBMC. The TLR4 reporter cell line was used to specifically assess the stimulatory activities of the different lipid A structures (45), whereas *in vitro* cytokine release from PBMC tests the stimulatory potential of a substance in the context of all TLRs and thus has been used to estimate human responses and to predict the safety of vaccines (38). In the TLR4 reporter cell line, we could detect no residual TLR4 activity of the penta-acylated lipid A in *S. sonnei* Δ*htrB*, indicated by a 60,000× increase in the amount of GMMA (500 ng/ml), required to give similar activation compared with GMMA with WT lipid A (0.008 ng/ml). GMMA from both *S. sonnei* and *S. flexneri* 2a Δ*msbB* mutants gave similar TLR4 activation at comparable GMMA concentrations (4.7 and 4.6 ng/ml). Although this required 600× more GMMA compared with GMMA with WT lipid A, the amount was 100× less compared with GMMA from the *S. sonnei* Δ*htrB* with penta-acyl lipid A, showing that the Δ*msbB* penta-acyl lipid A retains some residual ability to activate human TLR4. Although the magnitude of difference in TLR4 activation between the *Ss*_−p − OAg_ Δ*htrB* and the Δ*msbB* GMMA with penta-acylated lipid A was unexpected, minor modifications in penta-acylated lipid A have been shown to affect TLR4 stimulation (46). In *Sf*2a_−p − OAg_ Δ*htrB* GMMA, the substitution of a lauroyl chain with the longer palmitoleoyl chain in hexa-acylated lipid A also led to approximately a 50× decrease in the ability to simulate human TLR4 (0.4 ng/ml) compared with GMMA with WT lipid A. The mass spectrum showed a mixture of hexa-, penta-, and tetra-acylated lipid A, of which the palmitoleoylated hexa-acylated peak was by far the dominant peak present. Although the relative height of the peaks in this experiment is not necessarily strictly proportional to the abundance in the GMMA, it seems highly unlikely that a 50× reduction in activation could be due to the decrease in percentage of the hexa-acylated by the presence of these lower sized peaks but that the reduction of TLR4 stimulation is rather linked to the difference of the acyl chain composition. In PBMC, GMMA without lipid A modification predominantly stimulated the pro-inflammatory cytokines IL-6, TNF-α, IL-1β, and IL-8 as observed previously for *Neisseria meningitidis* native outer membrane vesicles (47). All GMMA with lipid A modification showed a marked decrease of cytokine release compared with GMMA with WT lipid A. IL-6 release was chosen for detailed comparison of the relative ability of GMMA to elicit cytokine release due to its role in fever pathogenesis (48). In contrast to the TLR4-specific assay, the difference in IL-6 stimulation by *S. sonnei* Δ*htrB* GMMA and *S. sonnei* or *S. flexneri* 2a Δ*msbB* GMMA was very small, only ∼3-fold (*S. sonnei* Δ*htrB* 800-fold and Δ*msbB* 300-fold), compared with the 100-fold difference in TLR4 activation (*S. sonnei* Δ*htrB* 60,000-fold and Δ*msbB* 600-fold). This small difference is in accordance with the presence of additional TLR being stimulated by GMMA. It indicates that the decrease of GMMA reactogenicity obtained by either form of penta-acylated lipid A is close to the maximum decrease of GMMA reactogenicity achievable by lipid A modification, when assayed in a complex system as PBMC that is more relevant for estimating responses in humans (38). Based on the large reduction of *in vitro* cytokine release from PBMC, an *S. sonnei* Δ*htrB* GMMA was chosen as a first candidate for vaccine development. Phase 1 clinical trials are currently underway and will give an important indication of the tolerability of GMMA from these constructs.

To characterize which TLR receptors contribute to the residual activity of GMMA, TLR blocking experiments in PBMC were performed. We demonstrated that the remaining activity of GMMA with penta-acylated lipid A was predominantly due to TLR2 activation. With GMMA from *Ss*_−p−OAg_ Δ*htrB,* no effect from TLR4 blocking was observed, whereas GMMA resulting from a *msbB* deletion retained detectable TLR4 activity in line with the results of the TLR4-specific assay. However, the induction of IL-6 via lipid A by the Δ*msbB* GMMA that was dependent on TLR4 was smaller than the induction via TLR2. In contrast, GMMA from *S. flexneri* 2a Δ*htrB* with palmitoleoylated hexa-acylated lipid A primarily stimulated TLR4. Further reduction of reactogenicity of GMMA from *Ss*_−p − OAg_ Δ*htrB* and the Δ*msbB* strains would require measures to reduce TLR2 activation. However, native outer membrane vesicles from *N. meningitidis* group B Δ*lpxL1* strain with penta-acylated lipid A (49) have been shown to be safe in Phase I clinical trials (50). Thus, although *N. meningitidis* and *Shigella* are not closely related and *e.g.* have different LPS structures (49) modification of TLR2 activators is not expected to be required.

The broad aim of this study was to examine ways of reducing the reactogenicity of GMMA to make them suitable for use as a human vaccine, but it resulted in a surprisingly complex outcome. Deleting the *htrB* gene from *S. sonnei* or the *msbB* gene from *S. sonnei* and *S. flexneri* 2a resulted in GMMA with a penta-acyl lipid A with a marked reduction in induction of inflammatory cytokines from human PMBC for which the residual activity is probably due to non-lipid A-related TLR2 activation. In contrast, compensatory palmitoleoylation in the *S. flexneri* 2a Δ*htrB* GMMA results in retained TLR4 activation. Although the level of reduction of GMMA reactogenicity required for an acceptable vaccine will depend on the dose required to give a strong immune response, which can only be determined in clinical trials, the data suggest that the use of GMMA as vaccines will likely require lipid A penta-acylation and that GMMA with palmitoleoylated hexa-acylated LPS are less likely to result in a useful vaccine.

## Footnotes

3 F. Citiulo, unpublished results.

## Footnotes

2 The abbreviations used are:

GMMA: generalized modules for membrane antigen
PBMC: peripheral blood mononuclear cell
TLR: Toll-like receptor
KDO: 2-keto-3-deoxyoctonate
OAg: O antigen.
